# Artificial Intelligence Performance in Image-Based Cancer Identification: Umbrella Review of Systematic Reviews

**DOI:** 10.2196/53567

**Published:** 2025-04-01

**Authors:** He-Li Xu, Ting-Ting Gong, Xin-Jian Song, Qian Chen, Qi Bao, Wei Yao, Meng-Meng Xie, Chen Li, Marcin Grzegorzek, Yu Shi, Hong-Zan Sun, Xiao-Han Li, Yu-Hong Zhao, Song Gao, Qi-Jun Wu

**Affiliations:** 1 Department of Clinical Epidemiology Shengjing Hospital of China Medical University Shenyang China; 2 Clinical Research Center Shengjing Hospital of China Medical University Shenyang China; 3 Liaoning Key Laboratory of Precision Medical Research on Major Chronic Disease Shengjing Hospital of China Medical University Shenyang China; 4 Department of Obstetrics and Gynecology Shengjing Hospital of China Medical University Shenyang China; 5 Department of Epidemiology School of Public Health China Medical University Shenyang China; 6 Microscopic Image and Medical Image Analysis Group College of Medicine and Biological Information Engineering Northeastern University Shenyang China; 7 Institute for Medical Informatics University of Luebeck Luebeck Germany; 8 Department of Radiology Shengjing Hospital of China Medical University Shenyang China; 9 Department of Pathology Shengjing Hospital of China Medical University Shenyang China; 10 Department of Clinical Epidemiology Shengjing Hospital of China Medical University Shenyang, Liaoning China; 11 NHC Key Laboratory of Advanced Reproductive Medicine and Fertility (China Medical University) National Health Commission Shenyang China

**Keywords:** artificial intelligence, biomedical imaging, cancer diagnosis, meta-analysis, systematic review, umbrella review

## Abstract

**Background:**

Artificial intelligence (AI) has the potential to transform cancer diagnosis, ultimately leading to better patient outcomes.

**Objective:**

We performed an umbrella review to summarize and critically evaluate the evidence for the AI-based imaging diagnosis of cancers.

**Methods:**

PubMed, Embase, Web of Science, Cochrane, and IEEE databases were searched for relevant systematic reviews from inception to June 19, 2024. Two independent investigators abstracted data and assessed the quality of evidence, using the Joanna Briggs Institute (JBI) Critical Appraisal Checklist for Systematic Reviews and Research Syntheses. We further assessed the quality of evidence in each meta-analysis by applying the Grading of Recommendations, Assessment, Development, and Evaluation (GRADE) criteria. Diagnostic performance data were synthesized narratively.

**Results:**

In a comprehensive analysis of 158 included studies evaluating the performance of AI algorithms in noninvasive imaging diagnosis across 8 major human system cancers, the accuracy of the classifiers for central nervous system cancers varied widely (ranging from 48% to 100%). Similarities were observed in the diagnostic performance for cancers of the head and neck, respiratory system, digestive system, urinary system, female-related systems, skin, and other sites. Most meta-analyses demonstrated positive summary performance. For instance, 9 reviews meta-analyzed sensitivity and specificity for esophageal cancer, showing ranges of 90%-95% and 80%-93.8%, respectively. In the case of breast cancer detection, 8 reviews calculated the pooled sensitivity and specificity within the ranges of 75.4%-92% and 83%-90.6%, respectively. Four meta-analyses reported the ranges of sensitivity and specificity in ovarian cancer, and both were 75%-94%. Notably, in lung cancer, the pooled specificity was relatively low, primarily distributed between 65% and 80%. Furthermore, 80.4% (127/158) of the included studies were of high quality according to the JBI Critical Appraisal Checklist, with the remaining studies classified as medium quality. The GRADE assessment indicated that the overall quality of the evidence was moderate to low.

**Conclusions:**

Although AI shows great potential for achieving accelerated, accurate, and more objective diagnoses of multiple cancers, there are still hurdles to overcome before its implementation in clinical settings. The present findings highlight that a concerted effort from the research community, clinicians, and policymakers is required to overcome existing hurdles and translate this potential into improved patient outcomes and health care delivery.

**Trial Registration:**

PROSPERO CRD42022364278; https://www.crd.york.ac.uk/PROSPERO/view/CRD42022364278

## Introduction

Globally, cancer accounted for 9.7 million deaths in 2022, and it accounts for nearly 1 in 6 deaths, based on updated estimates from the International Agency for Research on Cancer [1]. It is estimated that the global annual number of new cancer cases will increase to 35 million by 2050, causing a major global burden that varies markedly across countries and territories [1,2]. Due to its disordered growth, unlimited proliferation, and easy metastasis, patients at the terminal stage of cancer show a high probability of death [3]. The sensitive and accurate detection of cancers is critical to realizing timely and effective treatment, which can improve the survival rate of patients and reduce their pain [4].

In modern clinical practice, biomedical imaging systems represent one of the main pillars of comprehensive cancer diagnosis and grading [5]. Traditionally, pathological analysis has been considered essential to determine the malignancy of a tumor as it involves image analysis at the cellular level [6]. Additionally, noninvasive imaging techniques, such as ultrasound (US), computed tomography (CT), magnetic resonance imaging (MRI), and positron emission tomography (PET), have been popularly employed for tumor diagnosis [7]. However, biomedical imaging assessment most commonly relies upon visual evaluation, which is a subjective, susceptible, time-consuming, and labor-intensive process [8]. The interpretation of imaging findings may be augmented by advanced computational analyses [9]. With the high rate of production of images and the increasing reliance on images, the use of artificial intelligence (AI) to assist in imaging diagnosis has been a growing trend and an active research field [10,11]. Computer-aided diagnosis systems for medical imaging involving AI could help to overcome the mismatch between the increasing number of images and the capacity of available specialists [12].

Over the past decade, AI has made substantial strides in medical imaging, allowing machines to better interpret complex data [9]. In simple terms, AI excels at recognizing complex patterns in images and thus offers the opportunity to transform image assessment from a purely qualitative and subjective task to one that is quantifiable and effortlessly reproducible [9,13]. This property of AI extends its applicability to accurately detecting cancer from medical images, thereby holding the potential to unburden clinicians from repetitive tasks [14]. In the past, numerous studies have been published on AI-based imaging analyses in the diagnosis of different cancers. Advances in the use of AI for cancer diagnostic research are on the rise, with multiple examples, including the successful classification of dermoscopy images [15], the interpretation of mammograms for breast cancer screening [16], and the image-based differentiation between ovarian cancer and its benign imitators [17]. However, previous efforts to systematically appraise the evidence have been focused on a single tumor.

To assimilate the vast amount of research available and accelerate clinical transformation, we conducted an overview of systematic reviews for the purpose of systematically synthesizing previously published evidence on the performance of AI algorithms in the noninvasive imaging diagnosis of cancer.

## Methods

### Guidelines and Registration

This umbrella review was performed in line with the PRISMA (Preferred Reporting Items for Systematic Reviews and Meta-Analyses) guidelines [18] and Joanna Briggs Institute (JBI) guidelines (Multimedia Appendix 1) [19]. The predefined protocol was registered in the international open-access Prospective Register of Systematic Reviews (PROSPERO; CRD42022364278).

### Data Sources and Search Strategy

The present systematic literature search was completed using the following databases: PubMed, Embase, Web of Science, Cochrane, and IEEE. The search terms were as follows: (“artificial intelligence” OR “machine learning” OR “deep learning” OR “neural network”) AND (“carcinoma” OR “tumor” OR “cancer” OR “neoplas*” OR “maligna*”) AND (“meta-analysis” OR “systematic review”). The search was limited to articles written in English and published from inception to June 19, 2024. For the full search strategy, please see Multimedia Appendix 2.

### Eligibility Criteria

All systematic reviews with or without a meta-analysis that examined the noninvasive imaging diagnostic performance of AI technologies for cancers were eligible for inclusion. We also included reviews that had a systematic strategy for the literature search, even if the authors did not specify the article type [20]. Studies were excluded if they (1) focused on AI-based approaches for predicting the outcomes of interventions or the prognosis of cancers; (2) did not show at least one of the following measures of classifier performance: accuracy, sensitivity, specificity, or area under the curve (AUC); (3) did not involve noninvasive images, a systematic review, or interested outcomes; (4) were primary studies, scoping reviews, literature reviews, rapid reviews, criterial reviews, and other types of reviews; and (5) were conference abstracts and posters, commentaries, preprints, proposals, and editorials.

### Selection Process

Following the removal of duplicates, 2 investigators (TTG and HLX) independently screened the titles and abstracts to identify potentially relevant articles. The full texts of these articles were then assessed for eligibility based on the inclusion and exclusion criteria. Disagreement among the 2 reviewers was resolved by discussion with a third reviewer (XJS).

### Data Extraction

Two investigators (XJS and QB) independently extracted valuable data, using a predefined data extraction sheet. The following data were extracted: (1) characteristics of eligible reviews, including first author, year of publication, country, cancer site, registered protocol (yes or no), followed guidelines, research question, database searched, language restrictions, numbers of retrieved studies and included studies, number of reviewers (study selection, data extraction, and quality assessment), quality assessment tool, and meta-analysis (yes or no); (2) features of models in the included reviews, including imaging modalities, dataset size, AI approach, classification algorithms, and type of validation; and (3) classifier performance in multiple system cancers, including accuracy (n), sensitivity (n), specificity (n), AUC (n), and *I*^2^. Points of divergence were resolved by discussion among 2 authors (HLX and TTG).

### Quality Assessment and the Certainty of Evidence

Currently, AMSTAR 2 is the most widely used tool for evaluating the quality of systematic review methodologies [21]. However, the revision was not intended to deal with the special requirements of diagnostic systematic reviews [21]. Therefore, the methodological quality of the included articles was rated using only the JBI Critical Appraisal Checklist for Systematic Reviews and Research Syntheses [19]. This process was done independently by 2 investigators (MMX and WY), with group discussion when necessary.

The Grading of Recommendations, Assessment, Development, and Evaluation (GRADE) is the established tool for assessing the overall certainty of evidence [21-23]. For this review, we examined the following GRADE domains: (1) risk of bias in the individual studies, (2) inconsistency, (3) indirectness, (4) imprecision, and (5) publication bias. Based on these criteria, the meta-analytical evidence was classified as high, moderate, low, or very low. High and moderate certainty in evidence means that it is very likely or probable that the true effect lies close to the estimated finding, and low or very low certainty means that we have little or very little confidence in the finding [24,25]. This assessment was conducted by 2 independent researchers (QC and QB), with discussion and agreement for any differences.

### Narrative Synthesis

Given the significant heterogeneity between the reviews in terms of AI classifiers, imaging modalities, characteristics of participants, settings of conducted studies, etc, a narrative-only summary was considered. Study findings will be presented in graphs in the same way as the syntheses are reported in the narrative text to facilitate the comparison of findings from each included study [26].

## Results

### Study Selection

**Figure 1:**
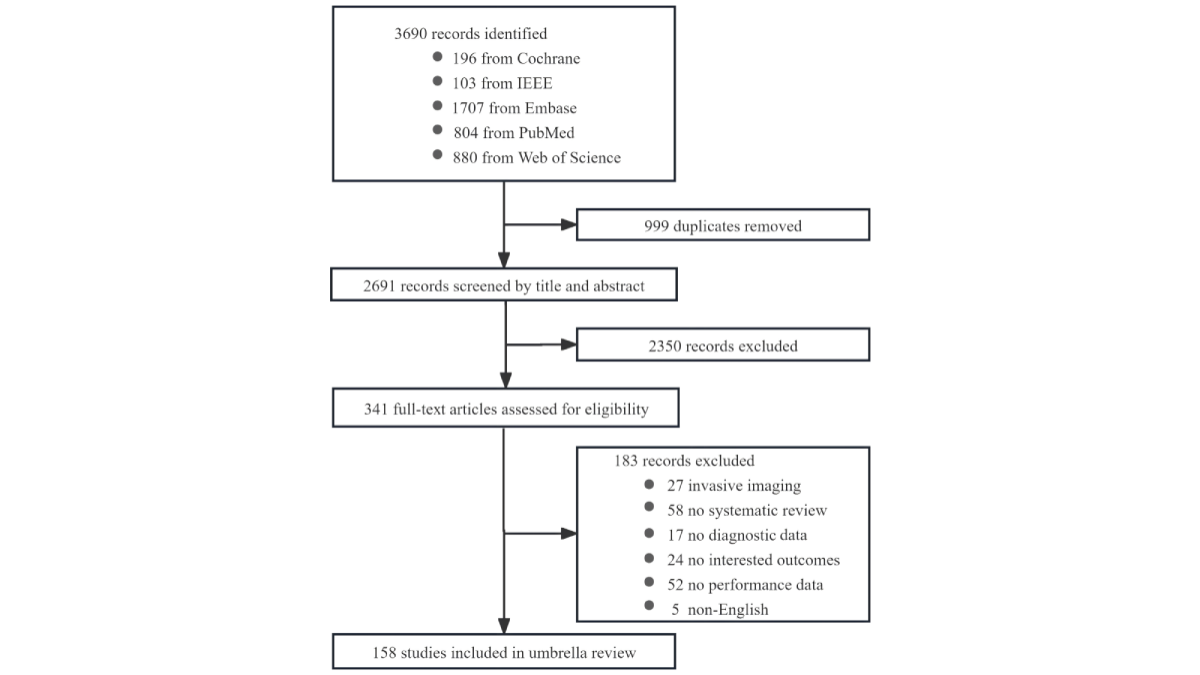
Flow diagram of the study selection process.

The detailed process for this umbrella review is presented in a schematic flowchart in Figure 1. The initial database search retrieved 3690 candidate publications, of which 2691 were screened following the removal of 999 duplicates. Based on the title and abstract, 2350 publications were excluded. The remaining 341 publications underwent a full-text examination to confirm eligibility. This resulted in a final total of 158 studies that met the inclusion criteria (Figure 1). The list of the excluded records during the process of full-text checking is provided in Multimedia Appendix 3.

### Study Characteristics

**Figure 2:**
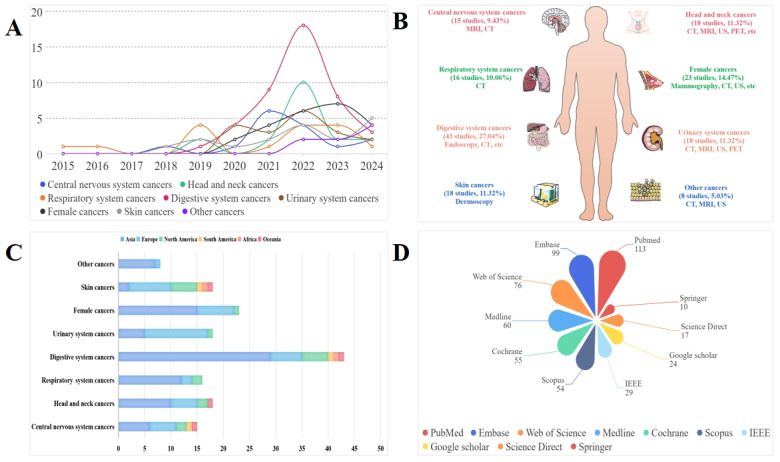
Characteristics of included systematic reviews by (A) year, (B) tumor site, (C) region, and (D) search database. CT: computed tomography; PET: positron emission tomography; MRI: magnetic resonance imaging; US: ultrasound.

**Figure 3:**
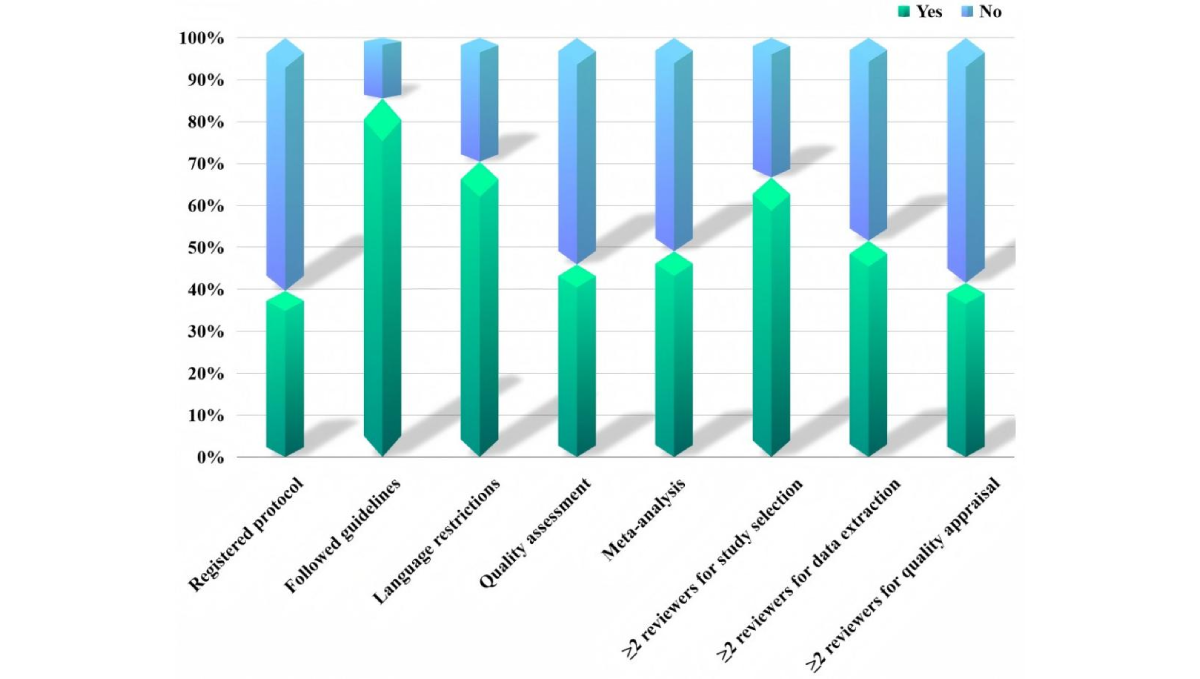
Characteristics of included systematic reviews by methodology.

The detailed characteristics of the included studies are presented in Figures 2 and 3 and Multimedia Appendices 4-6. More than half of the included studies (133/158, 84.2%) were published in 2021-2024 (Figure 2A). The included studies focused on cancers in the following 8 systems (Figure 2B): central nervous system (CNS) (n=15), head and neck (n=18), respiratory system (n=16), digestive system (n=43), urinary system (n=18), female-related systems (n=23), skin (n=18), and others (n=8). Among them, 1 review reported on both nervous system and respiratory system tumors. The included studies were conducted in 31 different countries, with half of them conducted in Asia (n=86) (Figure 2C). The most commonly searched database was PubMed (113/158, 71.5%) (Figure 2D). The number of retrieved and included studies in the meta-analyses or systematic reviews ranged from 279 to 117,464 and 3 to 32, respectively.

Different proportions of meta-analyses or systematic reviews had registered protocols, followed the PRISMA guidelines, restricted searches to English-language studies, and conducted quality assessment (Figure 3). Several criteria were mentioned for quality assessment, such as Quality Assessment of Diagnostic Accuracy Studies-2 (QUADAS-2), radiomics quality score (RQS), and Checklist for Artificial Intelligence in Medical Imaging (CLAIM). At least two independent reviewers carried out the study selection process, data extraction, and quality assessment in 106, 82, and 66 meta-analyses or systematic reviews, respectively. Seventy-seven meta-analyses or systematic reviews synthesized the data using quantitative analysis.

The studies included in the meta-analyses or systematic reviews used various imaging data to match the model. Among them, US, CT, and MRI were the most frequently adopted imaging approaches in most diseases. Moreover, dermoscopy in skin cancer, endoscopy in gastrointestinal tumors, and mammography in breast cancer were employed. A variety of algorithms were used in the included studies, and the most common ones were convolutional neural network (CNN) and support vector machine (SVM). Algorithms, including DenseNet, EfficientNet, RetinaNet, ImageNet, and Inception-v3, were frequently employed. Forty-nine meta-analyses or systematic reviews included articles that used both internal and external validation.

### Quality Appraisal Results

**Figure 4:**
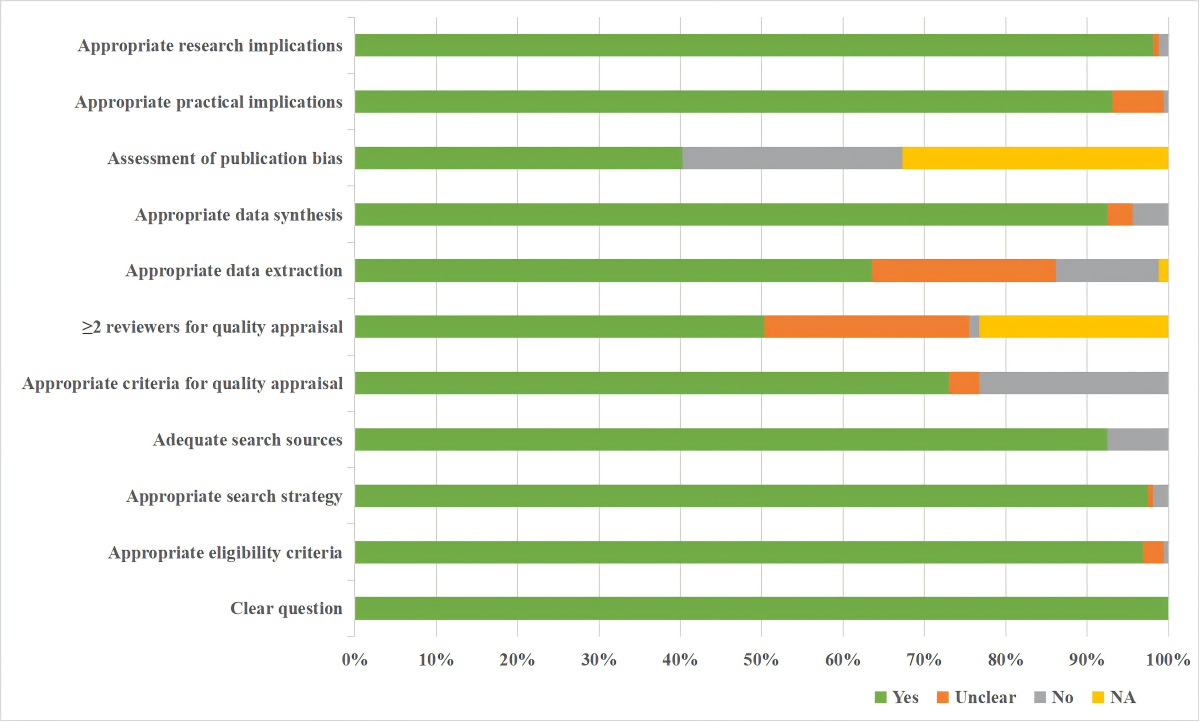
Quality of the included reviews assessed according to the Joanna Briggs Institute (JBI) tool. NA: not applicable.

The JBI critical appraisal results are summarized in Figure 4 and Multimedia Appendix 7. Overall, 80.4% (127/158) of the meta-analyses or systematic reviews were of high quality, and the rest were of medium quality. Out of 11 criteria, the minimum number of criteria met was 5 and the maximum was 11. Criteria 1-3, relating to clear questions, appropriate criteria, and appropriate search strategy, were met by the vast majority of included meta-analyses or systematic reviews.

### Grading of Evidence

**Figure 5:**
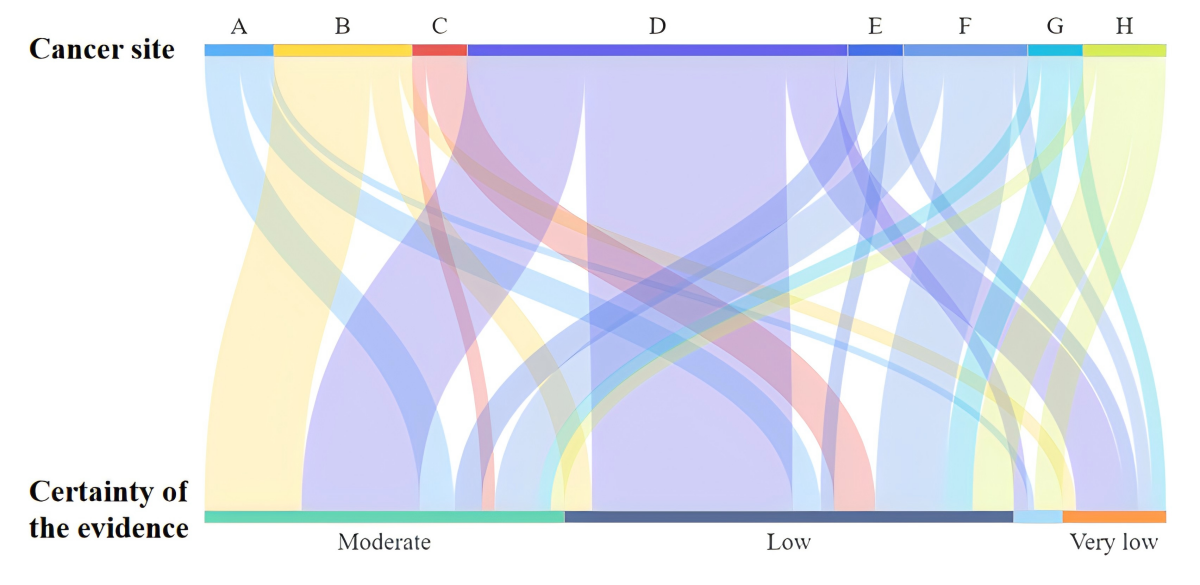
Evidence certainty of the included reviews assessed using the Grading of Recommendations, Assessment, Development, and Evaluations (GRADE) criteria. A: central nervous system cancers; B: head and neck cancers; C: respiratory system cancers; D: digestive system cancers; E: urinary system cancers; F: female cancers; G: skin cancers; H: other cancers.

The results of the GRADE assessment are presented in Multimedia Appendix 8 and Figure 5. Overall, 139 pieces of evidence met the conditions of the GRADE. The certainties of evidence were as follows: high, 5.0% (7/139); moderate, 37.4% (52/139); low, 46.8% (65/139); and very low, 10.8% (15/139).

### Summary of the Outcome

Overall, 158 meta-analyses or systematic reviews contained information on the diagnostic performance of AI for multisite cancers: brain, oral, nasopharyngeal, thyroid, lung, gastrointestinal, esophageal, liver, pancreas, renal, bladder, prostate, breast, cervical, ovarian, skin, chondrosarcoma, bone, adrenal, and soft tissue tumors. The performance of the AI models in diagnosing these cancers is presented in Multimedia Appendix 9.

#### CNS Cancers

Eight meta-analyses and 7 systematic reviews [27-41] evaluated the performance of AI algorithms in classifying CNS cancers, mainly using MRI, and 6 of these focused more specifically on the identification of gliomas. The accuracy, sensitivity, and specificity of the classifiers in the reviews ranged from 48% to 100%, 51% to 100%, and 40% to 97%, respectively. Among them, a recently published meta-analysis with a more comprehensive database search showed that the pooled sensitivity, specificity, and AUC were 94% (95% CI 91%-95%), 93% (95% CI 91%-95%), and 0.98 (95% CI 0.96-0.99), respectively [37]. However, the analysis showed an *I*^2^ of 97.6% for sensitivity and *I*^2^ of 96.7% for specificity, and other meta-analyses showed similar heterogeneity. Based on the JBI tool, 93% (14/15) of reviews were of high quality in CNS cancers. According to the GRADE criteria, 50% (5/10) of the evidence was rated as moderate.

#### Head and Neck Cancers

Eleven meta-analyses and 7 systematic reviews [42-59] summarized the performance of AI in using multiple imaging data to diagnose head and neck cancers, including oral, larynx, pharynx, and thyroid lesions. Several representative systematic reviews deserve to be highlighted. Rokhshad et al [44] reviewed deep learning (DL) applications for detecting head and neck cancer, using radiographic data. The accuracy, sensitivity, and specificity varied from 82.6% to 100%, 74% to 99.7%, and 66.6% to 90.1%, respectively. In a recently published meta-analysis of AI evaluating images of oral lesions, the pooled sensitivity and specificity were 88% (95% CI 87%-88%) and 80% (95% CI 80%-81%), respectively [47]. According to a meta-analysis of the largest number of included studies, the pooled sensitivity and specificity for radiomics in distinguishing thyroid nodules were 87% (95% CI 86%-87%) and 84% (95% CI 84%-85%), respectively [57]. In addition, the estimated sensitivity and specificity of the differential diagnosis between benign and malignant laryngeal lesions were 91% (95% CI 85%-97%) and 94% (95% CI 89%-100%), respectively [45]. With *I*^2^ ranges from 62% to 98.3%, the above pooled analyses revealed a significant variation between studies [44,45,47,48,50-52,54-57]. According to the GRADE criteria, 70% (14/20) of the evidence was judged as moderate.

#### Respiratory System Cancers

Six meta-analyses and 10 systematic reviews [33,60-74] examined the performance of AI in lung cancer assessment, mainly using CT. The results from 5 meta-analyses indicated that the pooled sensitivity was greater than 84%, while 1 reported a value of 77% [62]. Nevertheless, the pooled specificity was relatively low, mainly distributed around 65% to 80%. It has been suggested that AI-assisted CT diagnostic technology for the classification of pulmonary nodules as benign or malignant has good diagnostic performance, but its specificity needs to be improved. The GRADE criteria indicated 75% (6/8) low-quality evidence, suggesting that caution should be exercised when applying these findings in a clinical setting.

#### Digestive System Cancers

Forty-three meta-analyses or systematic reviews [75-117] explored the performance of AI in digestive system cancers, including cholangiocarcinoma (n=1), colorectal neoplasia (n=1), esophageal cancer (n=11), gastric cancer (n=19), hepatocellular carcinoma (n=4), and pancreatic cancer (n=7). Among these systematic reviews, 9 meta-analyzed sensitivity, specificity, and AUC and showed ranges of 90% to 95%, 80% to 93.8%, and 0.88 to 0.97, respectively, for esophageal cancer diagnostic performance [77-80,82-86]. For gastric cancer, 2 recently published meta-analyses showed aggregate AUC values greater than 0.94 [95,105]. Additionally, by using QUADAS-2, the included pooled studies were generally rated as high quality. Salehi et al [110] recently published meta-analyses related to hepatocellular carcinoma and reported that the pooled sensitivity and specificity for internally validated AI algorithms were 84% (95% CI 81%-87%) and 92% (95% CI 90%-94%), respectively, and those for externally validated AI algorithms were 85% (95% CI 78%-89%) and 84% (95% CI 72%-91%), respectively. They further found that the diagnostic performance of MRI images was slightly higher than that of CT and US images [110]. Five meta-analyses examined the performance of AI in diagnosing pancreatic cancer, using endoscopic US [111-113,115,116], reporting ranges of 90.4%-93% and 84%-95% for combined sensitivity and specificity, respectively. The evidence quality for the digestive system was classified according to the GRADE criteria as follows: high, 3% (2/55); medium, 31% (17/55); low, 53% (29/55); and very low, 13% (7/55).

#### Urinary System Cancers

Eighteen meta-analyses or systematic reviews [118-135] examined the performance of AI in urinary system cancers, using CT, PET, and MRI data. In a representative meta-analysis of bladder cancer [120], 8 studies with a total of 860 patients were included. The summary estimates for sensitivity and specificity in predicting bladder cancer were 82% (95% CI 77%-86%) and 81% (95% CI 76%-85%), respectively. More importantly, there was no relevant heterogeneity in diagnostic accuracy measures (*I*^2^=33% and 41% for sensitivity and specificity, respectively). In the systematic reviews of prostate cancer diagnosis [121-134], the accuracy, sensitivity, and specificity of the classifiers ranged from 29.7% to 100%, 22% to 100%, and 6% to 100%, respectively. Among them, a well-structured meta-analysis reported pooled sensitivity and specificity of 81.5% (95% CI 41%-99%) and 83% (95% CI 42%-99%), respectively. The calculated heterogeneity values for the pooled sensitivity and specificity were 84% and 79%, respectively (*P*<.001) [128]. The GRADE assessment revealed that 50% (4/8) of the evidence was of moderate quality, while the remainder had either low or very low quality.

#### Female Cancers

Twenty-three studies focused on female systemic tumors [17,136-157]. It is noteworthy that 15 meta-analyses or systematic reviews [136-150] examined the performance of AI in breast cancer detection. Eight of these pooled the results using meta-analysis, and the calculated pooled sensitivity and specificity ranged from 75.4% to 92% and 83% to 90.6%, respectively. It is worth mentioning that in the study by Oh et al [149], MRI demonstrated a sensitivity of 91% (95% CI 89%-92%) and specificity of 84% (95% CI 82%-86%), mammography-based radiomic features demonstrated a sensitivity of 79% (95% CI 76%-82%) and specificity of 81% (95% CI 79%-84%), and US-based analysis yielded a sensitivity of 92% (95% CI 90%-94%) and specificity of 85% (95% CI 83%-88%). The aforementioned studies exhibited similar diagnostic test accuracy for differentiating benign and malignant breast lesions via radiomic analysis. For ovarian cancer, 4 meta-analyses reported that the ranges of sensitivity and specificity were 75%-94% [17,154-156]. Furthermore, 2 meta-analyses performed subgroup analysis of imaging modalities, with AUC values of 0.94 and 0.95, 0.82 and 0.82, and 0.92 and 0.90 for US, CT, and MRI, respectively [17,154]. The GRADE evaluation indicated that 33% (6/18) of the evidence was of moderate quality. It is notable that this proportion implies a certain level of reliability in the presented findings but also suggests the need for further validation and refinement.

#### Skin Cancers

Four meta-analyses and 14 systematic reviews [10,158-174] explored the role of AI in skin cancer assessment and exhibited favorable performance. For instance, a newly published meta-analysis [172] found sensitivity and specificity values of 87% and 77.1%, respectively, for AI algorithms, and 79.8% and 73.6%, respectively, for all clinicians (overall). The differences were statistically significant. Moreover, pooled data from 70 studies presented the summary estimates of computer-aided diagnosis systems for melanoma, with a sensitivity of 74% (95% CI 66%-80%) and specificity of 84% (95% CI 79%-88%) [161]. The GRADE evaluation revealed that 75% of the evidence was of either low or very low quality. Admittedly, the included studies presented diverse methodologies and significant heterogeneity regarding the types of images included, the characteristics of the participants, and the methodology for presenting the results.

#### Other Cancers

Several systematic reviews have focused on some uncommon tumors. For instance, only 1 review evaluated the clinical value of radiomic analysis for chondrosarcoma, and the models for the differential diagnosis of chondrosarcoma, although showing good performance, were considered to have weak evidence [175]. A meta-analysis investigated the performance of CT-based radiomics in diagnosing malignant adrenal tumors [176] and showed an overall pooled AUC of 0.88 (95% CI 0.85-0.91). Dai et al [177] and Zhu et al [178] systematically evaluated the application value of radiomics and DL in the differential diagnosis of benign and malignant soft tissue tumors and demonstrated a pooled sensitivity of 84% and 84% and specificity of 88% and 63%, respectively. Two systematic reviews [179,180] evaluated the diagnostic performance of AI algorithms in the detection of bone malignancy, and the pooled sensitivity and specificity for AI algorithms in 1 meta-analysis were 84% (95% CI 75%-90%) and 91% (95% CI 83%-96%), respectively. In addition, Bai et al [181] reported the diagnostic performance of an AI model for the early detection of lymphoma for the first time, with a pooled sensitivity of 87% (95% CI 83%-91%), specificity of 94% (95% CI 92%-96%), and AUC of 0.97 (95% CI 0.95-0.98). 

## Discussion

**Principal Findings**

This umbrella review presents an overview of the AI-image techniques in the domain of cancer diagnosis, using the JBI tool and GRADE guidelines. We included 158 pertinent systematic reviews or meta-analyses involving 8 major human systems, and the GRADE assessment revealed that the overall quality of the evidence was moderate to very low. Although the diagnostic performance of the classifiers varied greatly, most meta-analyses showed positive summary performance. This study witnessed the outstanding performance of AI algorithms in analyzing tumor images, which can accurately identify the characteristics and subtle changes of tumors. However, this emerging field still faces many uncertainties and problems that need to be solved, requiring in-depth discussions with scientific and objective attitudes.

### Representativity of Study Objects

Ideally, the diagnostic approach should be explored in the setting of its intended use. In reality, numerous studies were not representative of the general population and community practice, with studies being performed in an experimental environment. For example, to date, all published data investigating radiomics in the field of pancreatic neuroendocrine tumors were mainly single-centered and retrospective studies [182]. The datasets largely included lesions from patients recruited in specialist clinical settings, and the applicability of these classifiers remains theoretical, making it more difficult to replicate the findings [183]. Meanwhile, the number of positive images and negative images in some included studies was significantly different, which was highlighted in a review on gastrointestinal cancer [92]. As suggested in recently published guidelines [88,184], a step forward needs to be achieved. Both current and future studies need to be constantly refined and externally validated in multicentric, large-sample, randomized clinical trials. In this regard, public access to large databases of clinical and radiological correlated data will be instrumental [182]. The creation of large public image datasets with images as representative as possible of the world’s people to avoid racial bias is a major task in this research field [171,185].

### Trends and Challenges of AI Technology

The variety of algorithms used in the assessed studies was high, and among them, CNN and SVM were the most widely used DL and machine learning methods in classification steps. SVM was typically used for limited data, while CNN was preferred for larger datasets. This distinction reflects the researcher’s expertise and preferences but is primarily driven by data constraints [186]. Even if high-performance models generate reasonable results, data from different training environments can contain various types of bias and noise and may not be reproduced again [159,187], and technique challenges remain to be settled [170]. The ability of clinicians to accept and trust the outputs of an algorithm, when the decision-making process is not apparent or comprehensible to them, may prove to be an obstacle to adoption [188]. A vivid metaphor is that the algorithm is mostly like a “black box” [189]. Acceptability to medical professionals and regulatory agencies may be increased if there is enhanced understanding as to how an algorithm arrives at its decision. Moving forward, further research into so called “explainable AI” [190] may provide the necessary transparency, trust, and accountability desired by the health care profession.

It should be discussed that a wide range of metrics was employed to report diagnostic performance in AI studies. We noticed that the evaluations were not performed based on a single procedure, and many studies used their own indicators. For example, some studies reported only 1 indicator; hence, it is not possible to know whether the study performed better in terms of other indicators [186]. There is no uniform standard and evaluation standard for the specific AI model design. In view of certain parameters being comprehensive and useful in clinical practice, it is an ideal research process to compare the accuracy, sensitivity, specificity, or AUC of some models with those of other methods and datasets in studies [100].

In the domain of tumor imaging diagnosis with the application of AI, several notable trends have emerged. Regarding AI technology trends, the continuous refinement and enhancement of DL algorithms, especially the advancement of CNN, have significantly improved the precision in analyzing features from tumor images. For instance, advanced architectures, such as ResNet and DenseNet, have achieved remarkable success in enhancing diagnostic accuracy [37,51,52]. Moreover, the use of transfer learning and pretrained models has become increasingly prevalent. By leveraging pretrained models on large-scale general image datasets like ImageNet and fine-tuning them for tumor imaging data, training time and data requirements have been considerably reduced [43]. In terms of target task categories, tumor detection has emerged as a critical objective, where AI techniques strive to identify early tumor indications from subtle image variations [87,114]. The classification and grading of tumors have also received significant attention, helping physicians in formulating more individualized treatment plans [191]. Additionally, in the process of literature screening, we found that comparing pre- and posttreatment imaging to determine whether the tumor has shrunk or remained stable, has become an important area of focus [192,193].

The emergence of generative AI and transformer-based technology has brought new possibilities. Generative AI can generate realistic tumor simulation images, providing a novel approach for expanding training data and aiding physicians in better comprehending tumor morphological changes [194]. Transformer-based technology excels in handling sequential data, such as time series in medical imaging, contributing to capturing the dynamic development of tumors [195,196]. When it comes to foundation models, the exploration of large-scale ones, such as large language models, in the medical field is on the rise. Integrating image and related clinical text information enables more comprehensive diagnostic support [197-199]. Multi-modal fusion foundation models are emerging, integrating information from multiple imaging modalities like CT, MRI, and PET to enhance diagnostic accuracy and reliability [200]. As mentioned above, the trends in AI for tumor imaging diagnosis are moving toward greater precision, comprehensiveness, personalization, and efficiency, offering new prospects for the detection and further precise treatment of tumors.

### AI, Clinicians, and Patients

According to the findings of this study, the AI systems in cancer detection follow 2 directions: (1) as concurrent assistants aiding clinicians in diagnosis and (2) as standalone systems providing an independent assessment. In the former case, the AI system is used as an aid during interpretation [201]. In particular, when using images for diagnosis, factors, such as physician fatigue, stress, and limited experience, may lead to neglected or misdiagnosed diseases. In contrast, AI can continuously provide reliable performance over a short period of time, has the potential to compensate for limited human capabilities, can prevent mistakes made by doctors in clinical practice, and can facilitate the training and education of less experienced endoscopists [116]. In the latter case, AI can play an important role in areas where the economy is underdeveloped and medical resources are relatively scarce. However, a major concern is the reproducibility, reliability, and usability of the diagnostic capability of AI, as false-negative results will lead to misdiagnosis and false-positive ones may result in overdiagnosis [159,202]. In this respect, AI may not completely replace doctors, and human beings and machines working together in harmony is an ideal state that can result in optimum performance.

As accuracy becomes more important, consolidating the information found in various forms of clinical data is vital in future clinical practice [96]. If purely based on image modeling, it is evident that the approach does not take into account all the information a clinician would rely on to evaluate a difficult examination [203]. The addition of clinical data, such as race, age, and gender, as inputs for classifiers may help to increase classification accuracy. This supplemental data could benefit clinicians in their decision-making [171]. Furthermore, AI has shown its advantages in computational power and learning capacity. An AI model is adept at integrating a lot of information from most data, which has the potential to reduce the workload of clinicians substantially, as it is difficult for them to integrate complex data manually [96]. These aspects should be included in future work.

### Multidisciplinary Cooperation

Processing and modeling medical images have traditionally represented complex tasks requiring multidisciplinary collaboration. Technically, the lack of a suitable information technology infrastructure in medical practices, the absence of high-quality curated data, and difficulties to access and exchange data are inhibiting the translation of integrated diagnostics into clinical routine and even research. Looking at the issue from a legal perspective, the sensitive issues of data privacy and security, patient consent, and autonomy must be fully considered. This means that from a legal perspective, data acquisition, storage, transfer, processing, and analysis will have to comply with all laws, regulations, and further legal requirements. In addition, the law and its interpretation and implementation must constantly adapt to the evolving state-of-the-art in technology [204,205]. We have observed a growing trend of clinical studies using commercial software for image processing [12]. The cost-effectiveness evaluation of AI in diagnosis is also warranted. Multidirectional interactions should be implemented among physicians, developers, legislators, and economists to foster multidisciplinary collaboration to tackle these challenges jointly [71]. When malpractice cases involving medical AI applications arise, multidisciplinary teams need to provide necessary suggestions [190].

### Quality Assessment Tools of the Included Studies

Multiple tools were employed in our included systematic reviews to comprehensively assess the quality of the included articles. The studies were evaluated by the RQS [206], CLAIM [207], Transparent Reporting of a Multivariable Prediction Model for Individual Prognosis or Diagnosis (TRIPOD) statement [208], Image Biomarker Standardization Initiative (IBSI) guideline [209], and QUADAS-2/AI tool [116,210]. It should be noted that each tool has its own focus. For example, the RQS does not take into account sample size or how the model actually performs but instead represents an evaluation of both how rigorous model development is and how impactful the study may be to the field [211]. The IBSI was able to produce and validate reference values for radiomics features. The TRIPOD is a similar example that aims to promote the transparent reporting of diagnostic accuracy model studies. There are suggestions from researchers that CLAIM may guide the update of the TRIPOD and RQS, because it not only includes general reporting criteria but also allows extra distinction of unique shortness in DL. Considering the overlapping items and high correlation between these tools, researchers can choose an appropriate one or a combination as the case may be. We are also confident that relevant organizations or scholars will continue to refine existing tools to better guide and evaluate relevant research.

### Strengths and Limitations

The strengths of this umbrella review include an exhaustive search of medical and engineering databases; assessment of methodological quality using the JBI tool; independent screening, quality assessment, and data extraction processes; and methodological rigor in the conduct of the review achieved by following the PRISMA guidelines. We also identified challenges that will need to be overcome for the technology to be implemented into daily clinical practice [94]. Umbrella reviews represent the highest level of evidence synthesis currently available and are becoming increasingly influential in biomedical literature [212]. Evidence from the umbrella review highlights the massive opportunity for AI image–based cancer detection. Remarkably, by identifying potential biases and methodological limitations, the information provided can raise the awareness of health care decision makers regarding the strengths and weaknesses of available AI diagnostic tools.

Several limitations of this review merit consideration. First, this review included a synthesis of evidence from existing systematic reviews or meta-analyses [116]. Therefore, cancers that were not systematically reviewed in the pre-existing literature were not included in this umbrella review. The lack of generalizability and the problem of out-of-distribution lesions, such as rare or more harmful disease categories, are limitations that are not addressed by current studies. Second, we could not examine the algorithms that were built without publication, such as those developed for private companies or for business usage [12]. Third, this umbrella review was limited by substantial variability in terms of the characteristics of the patients included, method quality, and study design, which precluded a full statistical analysis. Furthermore, the GRADE assessment revealed that the overall quality of nearly half of the evidence was low (47%). Since secondary research is highly dependent on the quality and availability of primary studies, both this umbrella review and previous systematic reviews were considerably restricted in improving the current understanding of cancer diagnosis.

### Conclusion

Conclusively, the main points discussed and investigated in this review can be summarized as follows. AI algorithms have shown remarkable efficacy in the noninvasive imaging diagnosis of tumors. Notwithstanding the progress, certain challenges persist. The data used in current AI models often have limitations in terms of quantity, quality, and diversity, which can compromise the model’s generalization ability. Additionally, the lack of transparency and interpretability of some AI systems poses difficulties for clinicians in understanding and trusting the diagnostic results. In the future, efforts should be directed toward enhancing data quality and diversity, developing more interpretable AI models, and conducting large-scale, multicenter clinical trials to validate the practical effectiveness of these technologies. While AI holds tremendous promise in noninvasive tumor imaging diagnosis, a concerted effort from the research community, clinicians, and policymakers is required to overcome the existing hurdles and translate this potential into improved patient outcomes and health care delivery.

## Appendix

Multimedia Appendix 1 PRISMA (Preferred Reporting Items for Systematic Reviews and Meta-Analyses) checklist.

Multimedia Appendix 2 Search terms and search strategy.

Multimedia Appendix 3 List of the excluded records during the process of full-text checking.

Multimedia Appendix 4 Metadata of the included reviews.

Multimedia Appendix 5 Search sources, search results, study selection, data extraction, quality assessment, and data synthesis in the included reviews.

Multimedia Appendix 6 Features of models in the included reviews.

Multimedia Appendix 7 Reviewer judgements about each quality criterion for each included review.

Multimedia Appendix 8 The results of the Grading of Recommendations, Assessment, Development, and Evaluations (GRADE) assessment of the evidence certainty of diagnostic accuracy regarding the association between artificial intelligence image systems and multisite cancers.

Multimedia Appendix 9 Classifier performance in differentiating multiple system cancers.

## Abbreviations

AI: artificial intelligence
AUC: area under the curve
CLAIM: Checklist for Artificial Intelligence in Medical Imaging
CNN: convolutional neural network
CNS: central nervous system
CT: computed tomography
DL: deep learning
GRADE: Grading of Recommendations, Assessment, Development, and Evaluation
IBSI: Image Biomarker Standardization Initiative
JBI: Joanna Briggs Institute
MRI: magnetic resonance imaging
PET: positron emission tomography
PRISMA: Preferred Reporting Items for Systematic Reviews and Meta-Analyses
QUADAS-2: Quality Assessment of Diagnostic Accuracy Studies-2
RQS: radiomics quality score
SVM: support vector machine
TRIPOD: Transparent Reporting of a Multivariable Prediction Model for Individual Prognosis or Diagnosis
US: ultrasound
